# Safety Evaluation of Porcine Bile Acids in Laying Hens: Effects on Laying Performance, Egg Quality, Blood Parameters, Organ Indexes, and Intestinal Development

**DOI:** 10.3389/fvets.2022.895831

**Published:** 2022-05-24

**Authors:** Bowen Yang, Shimeng Huang, Shupeng Li, Zhihua Feng, Guoxian Zhao, Qiugang Ma

**Affiliations:** ^1^College of Animal Science and Technology, Hebei Agricultural University, Baoding, China; ^2^State Key Laboratory of Animal Nutrition, College of Animal Science and Technology, China Agricultural University, Beijing, China

**Keywords:** porcine bile acids, laying hens, safety evaluation, blood parameters, histopathology

## Abstract

Bile acids (BAs) have long been known to facilitate digestion, transport, and absorption of lipids in the small intestine as well as regulate host lipid metabolic homeostasis. However, excessive BAs may lead to long-term damage to tissue. Also, it is unknown whether different levels of porcine BAs supplementation could improve performance, host metabolism, intestinal functions in laying hens. Hence, this study was aimed to investigate the potential effects of BAs addition on laying performance, egg quality, blood parameters, organ indexes, and intestinal histopathology of hens in the late phase. A total of 300 58-week-old Hy-line Gray hens were randomly divided into five groups which fed a basal diet (control) or basal diets supplemented with 60, 300, 600, and 3,000 mg/kg BAs for 56 days. Compared with the control group, no significant differences (*P* > 0.05) were observed in egg production, egg weight, ADFI, and FCR of hens in 60, 300, 600, and 3,000 mg/kg BAs groups. Dietary 60 mg/kg BAs supplementation resulted in a significant increase (*P* < 0.05) in egg mass. Meanwhile, no significant differences were observed in egg quality, including eggshell strength, eggshell thickness, albumen height, and Haugh unit among any treatment groups (*P* > 0.05). Dramatically, dietary 3,000 mg/kg BAs supplement decreased yolk color (*P* < 0.05). There was no significant difference in the blood parameters such as WBC, RBC, HGB, HCT, and PLT among any treatments. However, in 3,000 mg/kg BAs group, ovary coefficient was lower than the control (*P* < 0.05), and serum urea and creatinine were higher than the control (*P* < 0.05). Also, kidney and oviduct injury appeared in 3,000 mg/kg BAs group. These results indicated that a porcine BAs concentration of 3,000 mg/kg may cause harmful effects while 600 mg/kg was non-deleterious to laying hens after a daily administration for 56 days, namely that dietary supplement of up to 10 times the recommended dose of BAs was safely tolerated by laying hens.

## Introduction

Bile acids (BAs), specific products of cholesterol catabolism in the liver, are the principal component of human and animal bile (1). Some BAs synthesized in the liver enter the gallbladder for storage, while others enter the systemic circulation and will be transported into the kidneys and excreted with the urine (2). BAs are released from the gallbladder into the duodenum after eating to participate in lipid absorption. Then, some of BAs will be bio-transformed under the action of gut microbiota. At the same time, BAs also affect the gut microbial community and structure (3). Most of BAs are reabsorbed back to the liver at the end of the intestine to complete an enterohepatic circulation (4).

As a signal molecule, BAs regulate cholesterol and glucose metabolism (5–7), and promotes lipolysis (8). Previous studies have shown that oral chenodeoxycholic acid (CDCA) can reduce the serum triglyceride content of human patients with hypertriglyceridemia (9, 10). Also, CDCA was proved to inhibit cholesterol synthesis in humans (11). In recent years, new evidence has been provided that the administration of cholic acid (CA) and CDCA to mice increases the energy consumption of brown adipose tissue and prevents obesity and insulin resistance (8, 12). There is the fact that BAs play an important role as a signal molecule *in vivo* through their two important receptors FXR and TGR5 (13). CDCA up-regulates the expression of human peroxisome proliferator activated receptor alpha (PPARα) by activating FXR, thus regulating lipid metabolism (14). In the small intestine, BAs are the ligand of lipase, which play indispensable parts in the hydrolysis of lipids by lipase (15). Pancreatic lipase has a special structure that makes it function only after interfacial activation (16). BAs stabilize lipase at the water-oil interface and make it play a stable role in hydrolysis (17). Due to many important functions of BAs, more and more studies are centered on how to use exogenous BAs to regulate fat absorption and metabolism in different animal models. Bilz et al. (18) found that adding 0.1% CDCA in the high-fructose diet of hamsters reduced the production of hepatic *de novo* lipogenesis, and deceased the concentrations of plasma triglyceride and cholesterol. It is reported that supplementation of 60 mg/kg porcine BAs in the high-fat diet of broilers improved their growth performance and carcass quality (19). Ding et al. (20) supplemented BAs to the high-lipid diet of juvenile large yellow croakers and found that 300 mg/kg BAs supplement improved weight gain and reduced the fat content in liver.

However, owing to the particularity of the structure and properties of BAs, some studies demonstrated that BAs induced plasma membrane damage, thereby inducing oxidative stress, apoptosis, or inflammation (21, 22). For instance, cholestasis and cholestatic nephritis are caused by excessive accumulation of BAs in circulation (23). Generally, the accumulation and retention of hydrophobic BAs are the main cause of cholestatic diseases (23). Excessive BAs in circulation lead to endothelial injury in kidneys (24).

It is well-known that avian bile mainly consists of CDCA and CA, while hyodeoxycholic acid and CDCA are predominant in swine bile (25). Porcine BAs are considered as potential substitutes for BAs because of their low cost, easy extraction, well-defined composition, and chemical stability. Previous studies have shown that dietary supplement of 60–80 mg/kg porcine BAs improved the performance of broilers by increasing intestinal lipase activity (19, 26), and addition of BAs up to 600 mg/kg in broiler diets has been proved to be safe (27). But as far as we know, there is a lack of well-established research on supplementing porcine BAs to laying hens at present, especially on the safety. To evaluate the safety of BAs, the effects of high-dose BAs on performance, egg quality, blood parameters, organ indexes, and intestinal development of laying hens were evaluated in our study.

## Materials and Methods

### Reagents, Animals, and Experimental Design

The experimental BAs were produced by Shandong Longchang Animal Health Care Co., Ltd. (Dezhou, China). BAs used in this experiment were extracted from swine bile. According to the method as previously described (28), the contents of BAs in the product were determined by liquid chromatography-tandem mass spectrometry. The product contained hyocholic acid (HCA) 7.88%, hyodeoxycholic acid (HDCA) 70.23%, and CDCA 18.01%.

A total of 300 healthy 58-week-old Hy-Line Gray laying hens (1.50 ± 0.05 kg) were purchased from Beijing Deqingyuan Agricultural Technology Co., Ltd. (Beijing, China). Hens were randomly divided into five treatment groups. Each group contained six replicates and ten hens per replicate. According to our previous results, 60 mg/kg is the optimal dose of BAs on laying performance and crude fat utilization of laying hens (unpublished data). Other BAs doses were set as 5-fold (300 mg/kg BAs), 10-fold (600 mg/kg BAs), and 50-fold (3,000 mg/kg BAs) of the optimal dose. Therefore, corn-soybean meal-based diet was added with 0, 60, 300, 600, and 3,000 mg/kg BAs, respectively. In the present study, the intake of BAs was about 0, 4.125, 20.625, 41.250, and 206.250 mg/kg·BW. The experiment lasted 56 d. Layers were housed to habituate before the experiment for 7 d. The nutrient levels of the basal diet were formulated according to the Chicken Feeding Standards of China (NY/T33-2004) and the National Research Council (29) recommendations. The feed composition is shown in Table 1 and BAs content of each group is shown in Table 2. Nutrients in diet were analyzed using methods provided by AOAC (30) for crude protein (988.05), calcium (927.02), total phosphorus (995.11), and methionine and lysine (994.12). All hens were housed in environmentally controlled cages (45 × 45 × 45 cm) with two hens per cage. The room temperature and the relative humidity maintained at ~26°C and 65 ± 10%, respectively. All hens were provided with feed and water *ad libitum* with exposure to 16 h of light/d.

**Table 1:** Composition and nutrient levels of basal diet (as-fed basis).

**Ingredient (%)**
Corn	62.25
Soybean meal	26.00
Dicalcium phosphate	1.70
Limestone	8.20
Sodium chloride	0.30
50% choline chloride	0.10
DL-methionine	0.12
Soybean oil	1.00
Vitamin premix^a^	0.03
Mineral premix^b^	0.30
Total	100
**Nutrient^c^**
AME (Kcal/kg)	2,700.70
CP (%)	16.54
Digestible met (%)	0.38
Digestible lys (%)	0.78
Calcium (%)	3.60
Total *P* (%)	0.65
Non-phytate *P* (%)	0.39

*AME, avian metabolic energy; CP, crude protein*.

a *The vitamin premix supplied (per kilogram of diet): 8,000 IU of vitamin A, 3,600 IU of vitamin D_3_, 21 IU of vitamin E, 4.2 mg of vitamin K_3_, 3.0 mg of vitamin B_1_, 10.2 mg of vitamin B_2_, 0.9 mg of folic acid, 15 mg of calcium pantothenate, 45 mg of niacin, 5.4 mg of vitamin B_6_, 24 μg of vitamin B_12_, and 150 μg of biotin*.

b *The mineral premix provided (per kilogram of diet): 6.8 mg of Cu (CuSO_4_·5H_2_O), 66 mg of Fe (FeSO_4_·7H_2_O), 83 mg of Zn (ZnSO_4_·7H_2_O), 80 mg of Mn (MnSO_4_·H_2_O), 1.0 mg of I (KI) and 0.3 mg of Se (Na_2_SeO_3_)*.

c *Nutrient levels are analyzed values*.

**Table 2:** Bile acids content in each treatment diet.

**Items**	**Bile acids (mg/kg)**				
	**0**	**60**	**300**	**600**	**3,000**
HCA (mg/kg)	0.00	4.70	23.64	47.26	236.39
HDCA (mg/kg)	0.00	42.13	210.69	421.38	2,105.88
CDCA (mg/kg)	0.00	10.81	54.00	108.05	542.00

*HCA, hyocholic acid; HDCA, hyodeoxycholic acid; CDCA, chenodeoxycholic acid*.

### Laying Performance

Eggs are collected at a fixed time every single day. Egg production (EP) and egg weight (EW) were calculated every week. EP was the daily total number of eggs in each replicate divided by the number of hens of this replicate. The remaining feed weight was recorded every 7 days, and then the average daily feed intake (ADFI) was calculated. Egg mass (EM) = EP × EW. Feed conversion ratio (FCR) is expressed as the ratio of feed intake to egg mass and calculated weekly.

### Egg Quality

On the 28 and 56 d of the experiment, five eggs were randomly selected from each replicate for egg quality characteristics determination. Egg analyzer was used to determine the albumen height, Haugh unit, and yolk color. Egg Force Reader was used to determine the eggshell strength. Eggshell Thickness Tester was used to determine the eggshell thickness. The above three kinds of equipment were from Orka Food Technology Co., Ltd. (Ramat HaSharon, Israel). Eggshell thickness was the average value of blunt, middle, and sharp points on eggs. Percentage of yolk or eggshell were calculated as the percentage of whole egg weight.

### Sample Collection

On the last day of the experiment (56 d), two hens were randomly selected from each replicate for sample collection after 12 h fasting. Blood samples were taken through the wing vein of hens into 10 mL vacutainer tubes with or without anticoagulant (Greiner Bio-One GmbH, Frickenhausen, Germany). After standing at room temperature, blood in anticoagulant-free vacutainer tubes was centrifuged at 1,500 × *g* at 4°C for 15 min to prepare serum. Blood in vacutainer tubes with EDTA-K_2_ as the anticoagulant were determined immediately after collected.

The selected two hens were euthanized after blood collecting. Heart, liver, spleen, lung, kidney, pancreas, ovary, and oviduct were quickly taken out and weighed. Organ coefficient was expressed as the ratio of organ weight to live body weight. Portions of heart, liver, spleen, kidney, small intestines (about 0.5 cm of the same position of duodenum, jejunum, and ileum each bird), magnum of oviduct and ovary were taken and fixed in 4% paraformaldehyde solution for next determination.

### Clinical Blood Parameters Analysis

Blood routine indexes were determined by Automatic Blood Analyzer (Sysmex XN-1000, Sysmex Corp., Kobe, Japan) including white blood cell count (WBC), red blood cell count (RBC), hemoglobin (HGB), hematocrit (HCT), and platelet count (PLT). The following serum biochemical indexes were determined by Automatic Biochemical Analyzer (Hitachi 7600, Hitachi High-Technologies Corp., Tokyo, Japan): glutamate aminotransferase (ALT), aspartate aminotransferase (AST), alkaline phosphatase (ALP), total protein (TP), serum urea (UREA), and creatinine (CREA).

### Histopathology and Intestinal Morphology

After fixing with 4% paraformaldehyde solution for 24 h, tissue samples were dehydrated, cleared, and embedded in paraffin. Sections were stained with hematoxylin-eosin (H&E) and observed under a microscope and photographed. According to the results of microscopic examination, six visual fields were randomly selected from each section to score the histopathology of liver (31), heart (32), and kidney (33).

The intestinal mucosal damage was graded by Chiu's score (34) as follows: 0 = Normal mucosal villi. 1 = Gruenhagen's space appeared at the top of villi, capillary congestion. 2 = Extension of subepithelial space and the elevation of epithelial layer from lamina propria. 3 = Lots of epithelial cells shed and scattered around villi. Some villi have broken tips. 4 = Many villi fall off. Lamina propria and dilated capillaries exposed. Lamina propria cells increased. 5 = The lamina propria disintegrated. Hemorrhage and ulceration were observed. At the multiple of 10 × 10, the villus height (VH) and crypt depth (CD) of duodenum, jejunum, and ileum were measured under a microscope. Three visual fields were selected from each section, and five villi or crypts were selected from each visual field for measurement. Then the VH/CD ratio was calculated.

### Statistical Analysis

SAS 9.4 software (SAS Institute Inc., Cary, NC, USA) was used for data analysis. ANOVA in GLM procedure was used to analyze variance, and linear and quadratic *P*-values were also calculated using orthogonal contrasts. The level of statistical significance was set at *P* < 0.05. Tukey's test was used for *post-hoc* multiple comparisons. Results were shown as means. The overall standard error of mean (SEM) was given. The replicate served as the experimental unit for the analysis of laying performance and egg quality.

## Results

### Laying Performance

There was no significant difference among levels of BAs on EP, EW, ADFI, and FCR of laying hens (*P* > 0.05; Table 3). EM of hens in 60 mg/kg BAs group increased obviously compared to that of 0, 600 and 3,000 mg/kg BAs group (*P* < 0.05), while no significant difference existed between those of 60 and 300 mg/kg group (*P* > 0.05). In addition, there were no linear and quadratic significant changes in each index of laying performance.

**Table 3:** Effect of porcine bile acids on performance in laying hens^1^.

**Items**	**Bile acids (mg/kg)**					**SEM**	***P*** **-Value**		
	**0**	**60**	**300**	**600**	**3,000**		**ANOVA**	**Linear**	**Quadratic**
EP (%)	86.59	90.07	89.91	88.13	87.53	0.74	0.308	0.910	0.772
EW (g)	60.83	60.66	59.15	59.80	60.12	0.24	0.187	0.664	0.803
EM (g/bird/d)	52.67^b^	55.09^a^	54.74^ab^	52.70^b^	52.58^b^	0.37	0.040	0.602	0.531
ADFI (g/bird/d)	109.13	111.47	108.28	107.74	109.57	0.66	0.451	0.693	0.878
FCR (Feed/egg, g/g)	2.07	2.06	2.05	2.06	2.09	0.02	0.196	0.559	0.460

*EP, Hen-day egg production; EW, Average egg weight; EM, Egg mass; ADFI, Average daily feed intake; FCR, Feed conversion ratio*.

*^1^Data represent mean values of 6 replicates each treatment. SEM means standard error of the mean*.

a, b *Means within a row with no common superscripts differ significantly (P < 0.05)*.

### Egg Quality

Results of egg quality (Table 4) manifested that there was no remarkable effect on eggshell strength, eggshell thickness, albumen height, Haugh unit, yolk percentage, and eggshell percentage among treatment groups in both 28 and 56 d (*P* > 0.05). However, when the dose of BAs was 3,000 mg/kg, yolk color decreased in both 28 and 56 d (linear and quadratic, *P* < 0.05), whereas other doses of BAs did not affect the yolk color (*P* > 0.05).

**Table 4:** Effect of porcine bile acids on egg quality in laying hens^1^.

**Items**		**Bile acids (mg/kg)**					**SEM**	***P*** **-Value**		
	**Time (d)**	**0**	**60**	**300**	**600**	**3,000**		**ANOVA**	**Linear**	**Quadratic**
Eggshell strength (*N*)	28	33.72	35.17	33.61	33.72	32.72	0.71	0.882	0.550	0.836
	56	31.69	31.60	34.98	34.80	32.15	0.96	0.645	0.841	0.384
Eggshell percentage (%)	28	8.62	8.77	9.18	9.02	9.13	0.08	0.107	0.164	0.098
	56	8.91	9.08	8.96	8.66	8.81	0.07	0.439	0.269	0.398
Eggshell thickness (mm)	28	0.34	0.35	0.34	0.35	0.35	0.01	0.825	0.454	0.705
	56	0.37	0.39	0.40	0.40	0.39	0.01	0.111	0.627	0.158
Albumen height (mm)	28	5.94	6.39	6.29	6.11	6.63	0.14	0.594	0.250	0.497
	56	6.13	6.32	5.29	6.12	6.03	0.13	0.141	0.803	0.837
Haugh units	28	74.41	79.72	78.88	77.11	79.25	1.19	0.630	0.591	0.862
	56	77.24	78.69	68.88	76.58	77.46	1.27	0.119	0.928	0.762
Yolk color	28	4.82^a^	4.67^a^	4.71^a^	4.44^ab^	4.00^b^	0.07	<0.001	<0.001	<0.001
	56	4.44^a^	4.39^a^	4.19^ab^	4.19^ab^	3.83^b^	0.06	0.011	0.002	0.013
Yolk percentage (%)	28	26.83	26.86	26.76	26.48	25.91	0.18	0.403	0.028	0.088
	56	27.27	27.57	28.00	26.91	26.26	0.20	0.069	0.003	0.020

*^1^Data represent mean values of 6 replicates of 5 eggs each. SEM means standard error of the mean*.

a, b *Means within a row column with no common superscripts differ significantly (P < 0.05)*.

### Clinical Blood Parameters

High-dose BAs had no obvious effect on WBC, RBC, HGB, HCT and PLT in blood routine indexes of laying hens (*P* > 0.05; Table 5). The addition of high-dose BAs had no significant effect on ALT, AST, and TP in laying hens' serum (*P* > 0.05). But when it comes to ALP activity, the BAs supplementation of 600 mg/kg and 3,000 mg/kg significantly increased it (*P* < 0.05). And the contents of UREA and CREA in serum also increased substantially when the dose of BAs reached 3,000 mg/kg (*P* < 0.05).

**Table 5:** Effect of porcine bile acids on blood routine indexes and serum biochemical indexes of laying hens^1^.

**Items**	**Bile acids (mg/kg)**					**SEM**	***P*** **-Value**		
	**0**	**60**	**300**	**600**	**3,000**		**ANOVA**	**Linear**	**Quadratic**
WBC (10^9^/L)	147.53	149.53	147.54	149.46	146.19	0.84	0.770	0.438	0.659
RBC (10^12^/L)	2.60	2.58	2.56	2.63	2.61	0.03	0.449	0.239	0.466
HGB (g/L)	99.50	97.11	95.82	99.63	94.33	1.47	0.792	0.431	0.609
HCT (L/L)	0.23	0.22	0.22	0.23	0.21	0.01	0.631	0.244	0.402
PLT (10^9^/L)	34.83	31.50	32.75	33.88	33.67	1.52	0.248	0.862	0.831
ALT (U/L)	24.49	25.63	24.07	24.54	26.67	0.99	0.159	0.354	0.420
AST (U/L)	159.64	160.15	165.40	161.30	162.24	2.91	0.302	0.153	0.322
ALP (U/L)	34.58^b^	31.96^b^	42.55^ab^	48.81^a^	48.03^a^	1.88	0.007	0.569	0.491
TP (g/L)	50.54	53.40	50.71	49.92	54.11	1.45	0.861	0.638	0.840
UREA (mmol/L)	0.36^b^	0.38^b^	0.37^b^	0.44^b^	0.48^a^	0.01	0.027	0.003	0.007
CREA (μmol/L)	21.12^b^	23.69^b^	21.85^b^	26.49^b^	33.94^a^	1.01	<0.001	<0.001	<0.001

*WBC, white blood cell count; RBC, red blood cell count; HGB, hemoglobin; HCT, hematocrit; PLT, platelet count; ALT, glutamate aminotransferase; AST, aspartate aminotransferase; ALP, alkaline phosphatase; TP, total protein; CREA, creatinine*.

*^1^Data represent mean values of 12 hens each group. SEM means standard error of the mean*.

a, b *Means within a row column with no common superscripts differ significantly (P < 0.05)*.

### Relative Organ Weight and Histological Observations

BAs levels did not greatly affect heart, liver, spleen, lung, kidney, pancreas, and oviduct coefficient (*P* > 0.05; Table 6). But the ovary coefficient significantly decreased in 3,000 mg/kg BAs group (*P* < 0.01).

**Table 6:** Effect of porcine bile acids on organ coefficients in laying hens^1^.

**Items**	**Bile acids (mg/kg)**					**SEM**	***P*** **-Value**		
	**0**	**60**	**300**	**600**	**3,000**		**ANOVA**	**Linear**	**Quadratic**
Heart (%)	0.42	0.40	0.40	0.40	0.38	0.01	0.320	0.060	0.074
Liver (%)	2.17	2.04	2.00	2.17	1.98	0.05	0.701	0.954	0.190
Spleen (%)	0.12	0.10	0.11	0.10	0.12	0.01	0.301	0.371	0.542
Lung (%)	0.32	0.37	0.36	0.36	0.37	0.01	0.433	0.110	0.240
Kidney (%)	0.69	0.70	0.66	0.67	0.66	0.01	0.757	0.522	0.823
Pancreas (%)	0.26	0.22	0.25	0.23	0.24	0.01	0.225	0.744	0.789
Ovary (%)	2.92^a^	2.95^a^	3.03^a^	2.89^a^	2.43^b^	0.06	0.004	<0.001	<0.001
Oviduct (%)	4.54	4.35	4.21	4.46	4.38	0.11	0.873	0.890	0.992

*^1^Data represent the mean value of 6 replicates of 2 hens each. Organ coefficient is expressed as the ratio of organ weight to live body weight. SEM means standard error of the mean*.

a, b *Means within a row column with no common superscripts differ significantly (P < 0.05)*.

**Figure 1:**
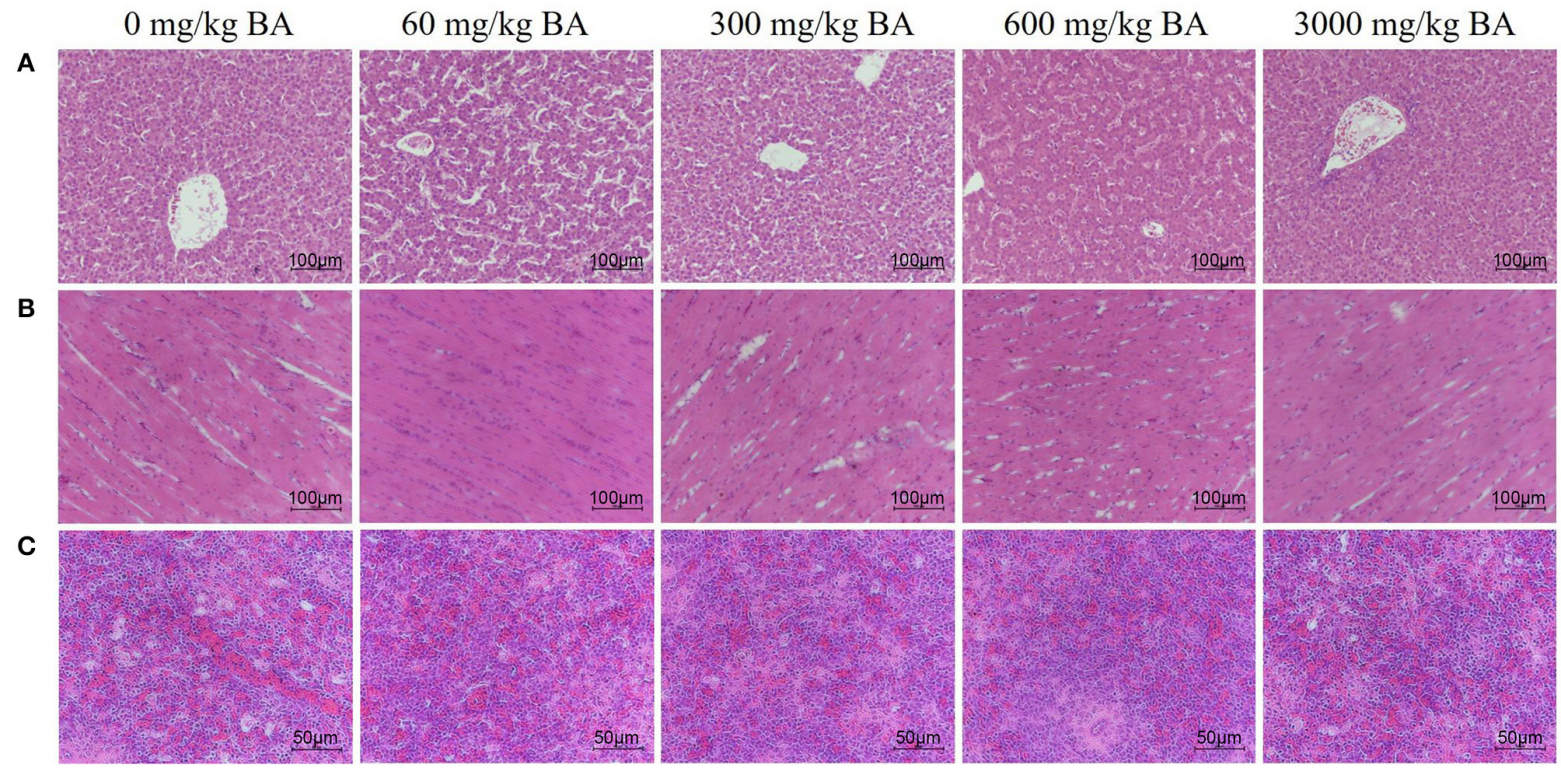
Effect of dietary porcine bile acids on organ morphology (heart, liver, and spleen) in laying hens. **(A)** H&E stained sections of liver. Scale bar, 100 μm. **(B)** H&E stained sections of heart. Scale bar, 100 μm. **(C)** H&E stained sections of spleen. Scale bar, 50 μm.

Supplementing of BAs did not obviously injured heart, liver, and spleen tissues of laying hens (Figures 1A–C). In the heart, the myocardial space was moderate and uniform, the myocardial fiber was complete, and there was no inflammatory cell infiltration and tissue proliferation. In the liver, the structure of hepatic sinusoid space and hepatocyte cord was clear and there was no inflammatory cell infiltration and lesions. In the spleen, no obvious apoptosis, tissue necrosis and inflammatory cell infiltration were found.

**Figure 2:**
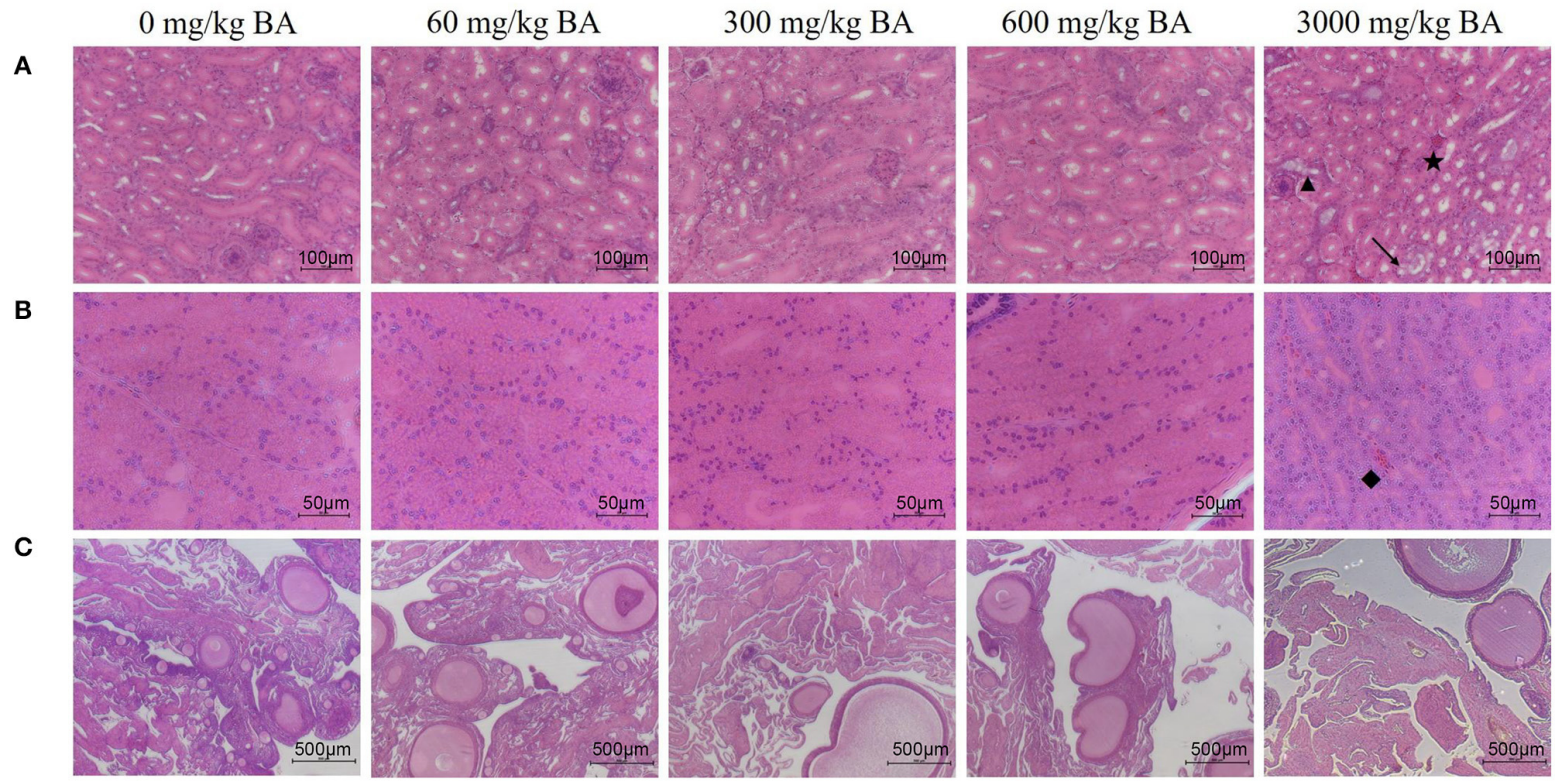
Effect of dietary porcine bile acids on organ morphology (kidney, oviduct, and ovary) in laying hens. **(A)** H&E stained sections of kidney. Scale bar, 100 μm. **(B)** H&E stained sections of oviduct. Scale bar, 50 μm. **(C)** H&E stained sections of ovary. Scale bar, 500 μm. → Indicates vacuolar degeneration of renal tubular epithelial cells. ⋆ Indicates renal interstitial edema and hemorrhage. ▴ Indicates dilation of renal tubular lumen. ♦ Indicates a large accumulation of red blood cells in the magnum tissues.

Symptoms of mild acute tubular injury can be found in hens of the treatment adding 3,000 mg/kg BAs evidenced by vacuolar degeneration, edema, hemorrhage, and dilation of renal tubular lumen (Figure 2A). Meanwhile, lots of red-staining substances were also observed in the magnum of oviduct of hens in the treatment adding 3,000 mg/kg BAs (black label in Figure 2B), demonstrating magnum was injured. However, there was no obvious ovarian injury observed in each BAs treatment (Figure 2C).

Correspondingly, no significant difference was observed in the histopathological scores of heart and liver (Table 7). But the scores of kidney increased in 3,000 mg/kg BAs group (*P* < 0.05).

**Table 7:** Effect of porcine bile acids on histopathology scores in laying hens^1^.

**Items**	**Bile acids (mg/kg)**					**SEM**	***P*** **-Value**		
	**0**	**60**	**300**	**600**	**3,000**		**ANOVA**	**Linear**	**Quadratic**
Heart	0.00	0.00	0.17	0.00	0.17	0.05	0.567	0.280	0.562
Liver	0.00	0.17	0.00	0.33	0.50	0.09	0.316	0.063	0.154
Kidney	0.00^b^	0.17^b^	0.17^b^	0.50^b^	2.00^a^	0.19	0.001	<0.001	<0.001

*^1^Data are means of scores of six visual field each section. SEM means standard error of the mean*.

a, b *Means within a row column with no common superscripts differ significantly (P < 0.05)*.

### Intestinal Morphology

**Figure 3:**
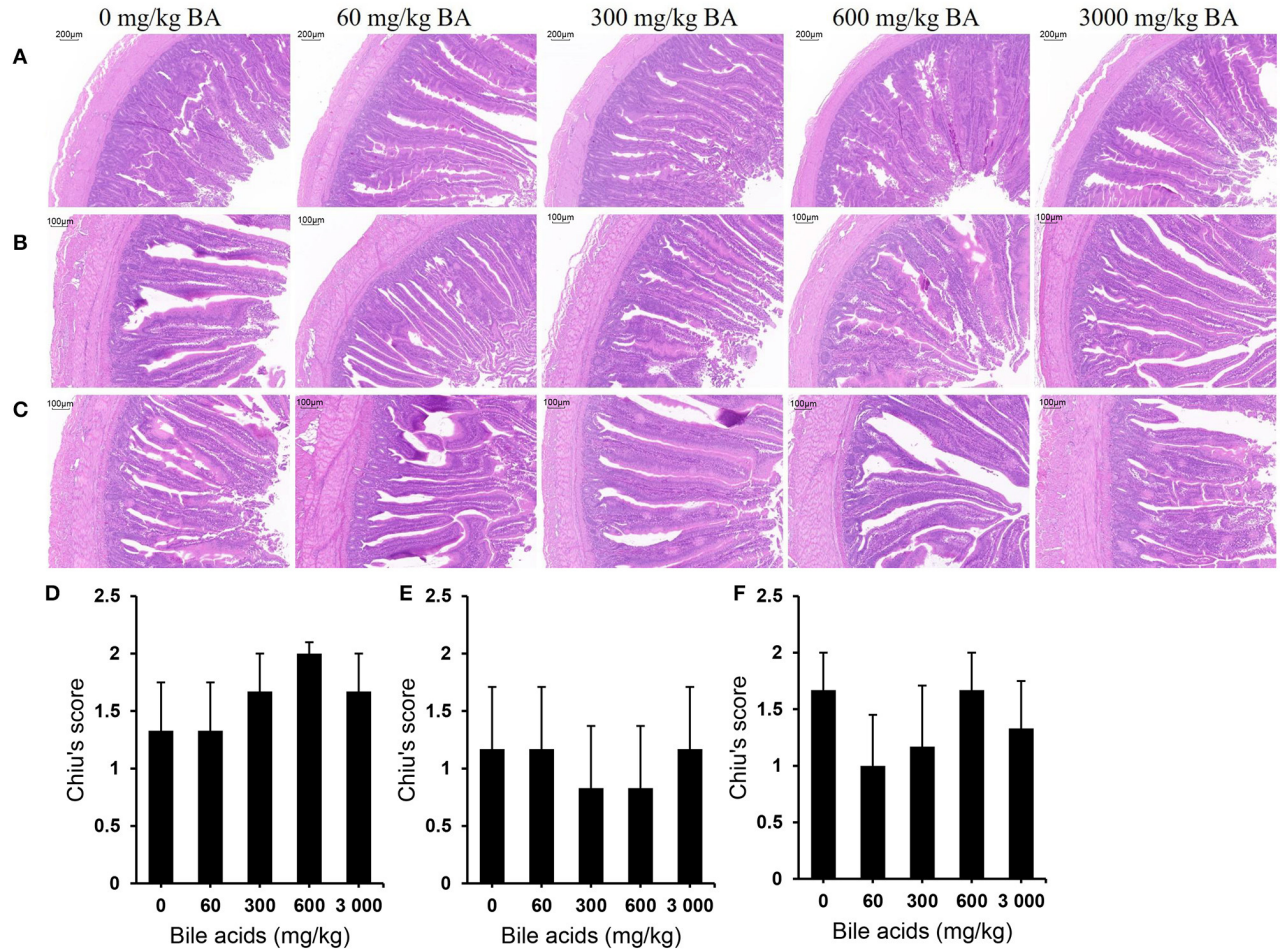
Effect of dietary porcine bile acids on intestinal morphology and Chiu's scores in laying hens. **(A)** H&E stained sections of duodenum. Scale bar, 200 μm. **(B)** H&E stained sections of jejunum. Scale bar, 100 μm. **(C)** H&E stained sections of ileum. Scale bar, 100 μm. **(D)** Chiu's score of duodenum. **(E)** Chiu's score of jejunum. **(F)** Chiu's score of ileum. **(D–F)** Each bar (mean ± SEM) represents the mean value of six replicates of two hens each.

No obvious injury was observed in all parts of small intestines (Figures 3A–C). In each segment of the small intestine, the muscularis and mucosal structures were intact, the villi and the crypt structure were normal, and there was no obvious congestion or hemorrhage. Likewise, the histological scores supported these results (Figures 3D–F). There was no significant difference in Chiu's scores among the treatments (*P* > 0.05). Table 8 exhibits the results of intestinal morphology. High-dose BAs had no significant effects on VH, CD, and VH/CD of laying hens (*P* > 0.05).

**Table 8:** Effect of porcine bile acids on intestinal development (villus height and crypt depth) in laying hens^1^.

**Items**		**Bile acids (mg/kg)**					**SEM**	***P*** **-value**		
		**0**	**60**	**300**	**600**	**3,000**		**ANOVA**	**Linear**	**Quadratic**
Duodenum	VH (μm)	1,496.18	1,574.98	1,493.98	1,483.72	1,541.14	14.93	0.255	0.602	0.385
	CD (μm)	248.64	266.48	257.88	253.560	249.24	3.73	0.575	0.423	0.730
	VH/CD	6.07	5.93	5.82	5.89	6.20	0.10	0.819	0.361	0.517
Jejunum	VH (μm)	816.92	850.10	851.40	832.10	861.36	6.55	0.210	0.146	0.356
	CD (μm)	125.92	132.28	124.48	125.54	132.34	1.82	0.489	0.324	0.408
	VH/CD	6.52	6.45	6.86	6.64	6.55	0.10	0.782	0.905	0.733
Ileum	VH (μm)	849.36	843.92	854.84	861.02	852.28	4.24	0.803	0.823	0.492
	CD (μm)	128.82	129.78	126.84	130.80	131.70	0.99	0.615	0.271	0.549
	VH/CD	6.61	6.50	6.74	6.59	6.48	0.06	0.609	0.378	0.574

*VH, villus height; CD, crypt depth*.

*^1^Data represent the mean value of three visual fields and five villi or crypts for each visual field each treatment*.

*SEM means standard error of the mean*.

## Discussion

BAs have always been the focus of medical and biological research because of their important roles. Previous studies found that BAs were ligand of lipase. In duodenum, pancreatic lipase hydrolyzes fat only by combining with BAs and co-lipase (15). Also, BAs make fat and water form fat-water mixture, thus expanding the contact surface between fat and lipase and accelerating the absorption of fat (35). With the enhancement of fat absorption capacity, egg production of laying hens will be also enhanced (36, 37). Thus, the function of BAs to promote fat absorption can be responsible for the increase of EM. Similarly, BAs have been shown to improve the performance of broilers by increasing the activity of intestinal lipases (19, 26). However, some *in vitro* experiments showed that excessive BAs were toxic to cells, and this toxicity may be exerted by reactive oxygen species (38). Overdose of BAs also induced plasma membrane damage and inflammation in rats or mice (21, 22). Furthermore, excessively high doses of BAs caused HepG2 cell apoptosis *in vitro* study (39). In the present study, no decline in laying performance in 3,000 mg/kg BAs group may be owing to the delicate balance of promotion of fat absorption and oxidative damage. Lai et al. (27) supplemented broilers with high-dose BAs and found that 400 mg/kg BAs had no adverse effect on the performance of broilers.

Egg yolk color is mainly derived from carotenoids in feed, especially zeaxanthin and lutein. Zeaxanthin and lutein are polar carotenoids that, unlike pro-vitamin A carotenoids, cannot be converted into vitamin A in the body (40). Competitive intake occurs when carotenoids enter the duodenum. Due to its polarity, lutein stays on the surface of fat droplets, while other carotenoids reside in the core of fat droplets (41). Large fat droplets in the duodenum are emulsified by our protagonist, BAs, into small fat droplets, forming micelles (42). The increase of micelles in the enteric cavity will reduce the transfer of lutein from the feed into the lipid phase. Moreover, if there are more phospholipids in the lutein micelles, absorption of lutein will also be inhibited (43). In general, BAs ascend the number of micelles in the enteric cavity, leading to the competitive absorption of polar carotenoids, and increases the solubilization of phospholipids, which may be responsible for the decline of yolk color. The present study also found that the change of yolk color showed the characteristics of linearity and quadratic, which may indicate that the improvement of fat dissolution is dose-dependent of BAs. Besides, most of the polar carotenoids in the liver are transported by low density lipoprotein (44). BAs up-regulate PPARα expression by activating FXR, thereby reducing hepatic apoC-III production and enhancing lipoprotein lipase activity (14, 45). Although a fraction of polar carotenoids transferred with very low density lipoprotein (yolk targeted) may not be affected by lipoprotein lipase (41), the enhancement of lipoprotein lipase can still affect the transfer of lutein and zeaxanthin.

Addition of high-dose BAs did not affect WBC, RBC, HGB, HCT, and PLT of laying hens. The similar results were also reported by Lai et al. (27). Blood cells may have sufficient tolerance to BAs. Likewise, Hwang et al. (46) proved that CA supplement did not affect RBC, HGB, and HCT in mice. Because high-dose BAs did not injure liver tissue, there were no significant changes in AST, ALT, and TP in serum of laying hens. The increase of ALP activity may not be due to tissue injury, but because CA or CDCA can promote the expression of ALP mRNA (47). UREA and CREA are end products of the metabolism that need to be excreted through the glomerulus. When the kidneys are seriously injured, the excretion of CREA and UREA will be blocked, resulting in the increase of CREA and UREA content. The present study showed that the addition of 3,000 mg/kg BAs increased the contents of serum UREA and CREA in laying hens, indicating that the kidneys of laying hens were seriously injured, which corresponded to the histological observation results. Cholestatic nephropathy is a kind of acute kidney injury disease caused by the pathological accumulation of BAs in the circulation. UREA and CREA in patients with cholestatic nephropathy usually higher than normal subjects (48). Besides, cholestatic jaundice, a disease caused by bile entering the circulation, also reduces the clearance rate of serum creatinine (49). In previous studies, BAs have been well-evaluated for its effect on reducing blood total cholesterol and triglycerides (11, 50). Our study verified the effect of oral porcine BAs on the serum biomarkers of tissue injury, providing a new perspective for the study of BAs. However, whether related gene expression in the liver or kidney, such as gluconeogenesis, is affected by ultra-high dose of BAs was not investigated, and this part requires further study.

Exogenous BAs play roles after entering the duodenum with the diet, and then most BAs will be reabsorbed at the end of the ileum and return to the liver through the enterohepatic circulation (2). Excessive BAs in the liver pass through the systemic circulation into kidneys (e.g., about 0.5 mg/d in a normal human body), then it will be excreted

with urine (4). Excessive concentration of BAs will increase renal burden and endothelial injury (24). Holmes (51) observed similar renal injury in patients with cholestatic nephropathy, in which enlarged cells entered the capsule space through the renal tubules, and pink substances (classic bile staining) were observed in the renal tubule and glomerular space. In the study of Fickert et al. (52), the enterohepatic circulation of BAs in bile-duct-ligated mice was blocked, BAs were discharged into the kidneys, resulting in renal injury, and a lot of pink substances (PAS-positive casts) were observed in distal tubules and collecting ducts. The above studies were similar to those of our study. The present study indicated that adding 3,000 mg/kg BAs in laying hen diet caused congestion and hemorrhage of magnum tissue and the decrease ovary coefficient. Although no obvious damage was observed on ovarian histology, the decrease in ovary coefficient could also indicate that 3,000 mg/kg BAs damaged ovarian tissue. This result may be due to the excessively high-dose of BAs causing cell membrane damage and cell apoptosis (21, 22). Magnum is the longest part of the oviduct of laying hens, with many folds. There are many glands in the magnum, which are related to the secretion of protein. Magnum and ovary injury will downgrade the reproductive capacity of laying hens. However, since BAs can improve fat absorption to some extent, this seemed to strike a delicate balance that could explain the absence of a decline in laying performance. The mechanism of high-dose BAs on oviduct injury needs to be further studied.

## Conclusions

In conclusion, the present study showed that even though a daily administration of BAs had a very favorable contribution to laying performance of laying hens, it may have some adverse effects once it exceeded a certain upper limit (3,000 mg/kg). However, using up to 600 mg/kg will be non-deleterious, which also suggested that porcine BAs would be a safe kind of promoter for laying hens as long as it was used at the recommended dose.

## Data Availability Statement

The raw data supporting the conclusions of this article will be made available by the authors, without undue reservation.

## Ethics Statement

The animal study was reviewed and approved by Institutional Animal Care and Use Committee of China Agricultural University.

## Author Contributions

BY performed the experiments and drafted the manuscript. BY, SL, SH, and QM carried out the statistical analysis. QM, SH, ZF, and GZ helped the revision of this manuscript. QM and GZ contributed to the supervision and guidance of the present study. All authors read and approved the final manuscript.

## Funding

This study was supported by the National Science Foundation of China (Grant No. 31930105), the Modern Agricultural Industry Technology System Construction Project of Hebei Province (Grant No. HBCT2018150203), and a special fund for China Agricultural Research System program (Grant No. CARS-40-K08).

## Conflict of Interest

The authors declare that the research was conducted in the absence of any commercial or financial relationships that could be construed as a potential conflict of interest.

## Publisher's Note

All claims expressed in this article are solely those of the authors and do not necessarily represent those of their affiliated organizations, or those of the publisher, the editors and the reviewers. Any product that may be evaluated in this article, or claim that may be made by its manufacturer, is not guaranteed or endorsed by the publisher.
